# Renting political violence: A political economy of rents, access and violence delegation

**DOI:** 10.1177/00223433251352676

**Published:** 2025-08-25

**Authors:** Maureen Fubara

**Affiliations:** Department of Political Science, University of Amsterdam, the Netherlands

**Keywords:** Armed groups, citizens, electoral violence, incumbents, rents

## Abstract

What explains variation in incumbents’ choice of political violence perpetrators? Incumbents often sponsor violence in elections but do not typically engage in it themselves, instead delegating violence to security forces, armed groups, party wings, or regular citizens. Existing theory poorly explains such variation, which has privileged incumbents’ incentives to plausibly deny their involvement. This article develops a theory centred around variation in electoral violence perpetrators. Focusing on the subnational level, I argue that variation in rents helps explain why some incumbents recruit armed groups while others rely on ordinary citizens. Incumbents with access to large rents can afford to hire costly yet effective armed groups. In contrast, those with limited rents recruit cheaper but less capable alternatives such as ordinary citizens. I use over one hundred interviews conducted with politicians, journalists, voters, civil society members and citizens in four Nigerian states, Lagos, Rivers, Plateau and Nasarawa, to probe the plausibility of the argument. I triangulate interview findings with newspapers and observer reports. Findings show that in Lagos and Rivers, incumbents hire and maintain armed groups such as transport workers and cult groups due to high rents, while those in the low-rents state of Nasarawa hire citizens to perpetrate violence. The study contributes to the literature on decentralization, joint production of political violence, and the resource curse.

## Introduction

In elections around the world, incumbents often resort to violence to maintain power, either to prevent defeat or consolidate control. Instead of engaging directly, they delegate violence to security forces, armed groups, violent party factions, or ordinary citizens (Berenschot, 2020; Brass, 2004; Klaus, 2020; Matanock and Staniland, 2018; Siddiqui, 2023; Staniland, 2021). Conventional wisdom suggests that this delegation allows plausible deniability, shielding incumbents from accountability (Acemoglu et al., 2013; Carey et al., 2015; Roessler, 2005). However, these explanations fail to address the conditions under which incumbents choose specific types of perpetrators. Empirical evidence reveals a diverse range of violent actors employed during campaigns. Politicians collaborate with armed groups (Matanock and Staniland, 2018; Siddiqui, 2023; Staniland, 2021), exploit ethnic networks (Berenschot, 2020; Brass, 2004; Wilkinson, 2004), or mobilize ordinary citizens (Klaus, 2020). However, incumbents’ choices among these perpetrators are poorly understood, leading to the following question: What explains variation in incumbents’ choice of political violence perpetrators?

My theoretical argument links variation in violence perpetrators to the uneven distribution of rents.^1^ I argue that while incumbents with access to extensive rents can hire and maintain costly but effective armed groups,^2^ those with limited rents have a weaker capacity to recruit armed groups, relying on ordinary citizens.^3^ Focusing on the subnational level, I empirically examine these expectations in Nigeria, a relevant case given its uneven rent distribution and history of violent elections. To probe the plausibility of the argument, I selected two high-rents states, Lagos and Rivers, and two low-rents states, Nasarawa and Plateau. The analysis is based on qualitative interviews conducted over eight months with local politicians, journalists, civil society members and voters, supplemented by newspapers and election observer reports.

The findings indicate that in Lagos and Rivers, incumbents leverage high rents to recruit and maintain armed groups, offering substantial financial rewards to secure their coercive capacities for demobilizing rivals. These relationships are long-term and sustained by significant financial investments. In contrast, in Nasarawa, low rents constrain incumbents to rely on ordinary citizens for electoral violence, establishing short-term relationships with minimal financial commitments. While findings in Plateau indicate that incumbents hire citizens for violence, I did not find sufficient evidence regarding the nature of their relationships after elections.

First, I contribute to work on the joint production of violence by incumbents and non-state actors. Prior work has primarily focused on incumbents’ collaboration with ordinary citizens (Berenschot, 2020; Boone, 2011; Klaus, 2020), outsourcing versus directly perpetrating violence (Siddiqui, 2023), and alliances with armed groups (Matanock and Staniland, 2018; Raleigh, 2016; Staniland, 2021; Turnbull, 2021a, 2021b), using different contexts. In contrast, my argument explains why and how subnational incumbents collaborate with distinct types of perpetrators within the same context, holding country factors constant.

Second, I contribute to the literature on the resource curse by linking rents and electoral violence. My article demonstrates that rents not only incentivize violence (Di John, 2007; Le Billon, 2001; Okoye and Taylor, 1999) but provide capacity for it. Moreover, I expand the applicability of the resource curse to the subnational level, showing that rent seeking is not limited to national incumbents and more serious forms of violence, such as civil war, but also applies to electoral violence organized by subnational incumbents.

Third, I contribute to the literature on citizen recruitment for electoral violence (Boone and Kriger, 2010; Klaus, 2020; Klaus and Mitchell, 2015; Wilkinson, 2004). While existing studies emphasize normative factors such as grievance narratives, collective marginalization and ethnicity, I shift the focus to the material conditions of citizens recruitment.

## Previous research

Elections in new and emerging democracies are commonly associated with violence (Birch et al., 2020). Research consistently shows that incumbents, more so than opposition candidates, have stronger motives, incentives and capacities to engage in electoral violence (Collier and Vicente, 2012; Frantzeskakis and Park, 2022; Hafner-Burton et al., 2014, 2018; Onapajo, 2014; Taylor et al., 2017; Turnbull, 2021a). For example, Taylor et al. (2017) find that incumbents were responsible for 80% of pre-election violence and 74% of post-election violence in sub-Saharan Africa between 1990 and 2008.

Unpacking incumbent motives, studies reveal that for vulnerable incumbents, violence is often instrumental in achieving strategic objectives such as expanding territorial control, eliminating opposition threats, and securing access to patronage resources (Burchard, 2020; Collier and Vicente, 2012; Frantzeskakis and Park, 2022; Hafner-Burton et al., 2014, 2018; Onapajo, 2014; Van Baalen, 2023; Von Borzyskowski and Kuhn, 2020). However, to avoid domestic and international costs, they often outsource violence to non-state actors such as rebels, militias, youth gangs, armed groups and ordinary citizens (Daxecker, 2014; Klaus, 2020; Matanock and Staniland, 2018; Raleigh, 2016; Rosenzweig, 2021; Siddiqui, 2023; Staniland, 2015a; Turnbull, 2021a, 2021b; Wilkinson, 2004). In this review, I focus on the two non-state actors: armed groups and ordinary citizens.

Incumbents rely on armed groups to demobilize rivals; these groups, in turn, leverage such relationships for political relevance, material benefits and power. For instance, incumbents use armed groups to fraudulently win elections and punish defectors, while these groups, in return, demand material and political support to secure their own electoral gains (Turnbull, 2021b, 2024). Staniland (2015b) finds that while ideological compatibility is important, it does not prevent incumbents from collaborating with incompatible groups when compatible ones are unavailable. However, these groups are not mere foot soldiers of incumbents; they exhibit agency by refusing to engage in violence or even participating in elections as candidates themselves (Matanock and Staniland, 2018; Turnbull, 2021b).

Regarding ordinary citizens, the literature mainly focuses on multi-ethnic contexts, exploring how contentious narratives around collective marginalization, political exclusion and economic inequalities shape their mobilization for violence (Boone and Kriger, 2010; Bulutgil and Prasad, 2020; Klaus, 2020; Klaus and Mitchell, 2015; Klaus and Turnbull, 2025). Politicians use such grievances rooted in salient ethnic cleavages to polarize citizens for violence (Boone, 2011; Brass, 2004; Wilkinson, 2004). While ethnicity is significant, there is limited understanding of other mechanisms through which citizens are recruited for violence. Linking the two literatures, Siddiqui (2023) argues that parties’ organizational capacity determines whether they engage in direct violence or delegate it to armed groups. However, while her theory offers valuable insights, it does not explain why in weak party contexts, parties with similar organizational capacity hire armed groups while others rely on citizens. Based on existing research, we do not know whether country-specific factors lead to citizen violence in some cases and armed group violence in others. I examine this variation at the subnational level, holding country-level factors constant.

## Rents, access and political violence delegation

My argument examines the variation in violence perpetrators, focusing on the conditions under which incumbents hire either armed groups or citizens to perpetrate electoral violence. Understanding where and why these actors differ offers insights into the contexts where intense electoral violence is likely to occur. I argue that this variation is shaped by material resources, specifically rents, rather than factors such as party identity, availability of perpetrators, ethnic polarization, or violent incentives. I define rents as unearned income from natural resources, foreign aid, or loans (Pérez Niño and Le Billon, 2014; Svensson, 2000). Unlike earned income, rents are likely to be mismanaged, given minimal fiscal accountability associated with their source of origin (Martin, 2014; Ross, 2001). I argue that incumbents’ access to rent significantly enhances or limits their capacity to hire distinct political violence perpetrators.

Although incumbents should ideally compete fairly, some resort to illicit violent strategies. This article highlights three reasons for incumbent-sponsored violence. First, vulnerable incumbents use violence to eliminate challengers and consolidate control (Collier and Vicente, 2012; Hafner-Burton et al., 2014). Second, the perks and privileges of office incentivize rent-seeking politicians to engage in violence (Okoye and Taylor, 1999; Taylor et al., 2017). Third, incumbents unwilling to concede defeat instigate violence to avoid acknowledging electoral losses (Fandos and Cochrane, 2021). While incumbents use licit and non-violent illicit strategies such as fraud (Bratton, 2008), they typically combine them with coercive ones such as violence, particularly in contexts where the associated costs are low.

Similar to their national counterparts, subnational incumbents also sponsor electoral violence, particularly in contexts plagued by the subnational resource curse. In such contexts, rents often undermine development, fostering weak institutions, corruption and violent rent seeking (Manzano and Gutiérrez, 2019; Paler, 2011). Hence, subnational incumbents leverage access to rents to organize violence (Fetzer and Kyburz, 2024). However, the uneven distribution of resources across subnational units (Harbers and Steele, 2020), I argue, creates disparities in the availability of rents, impacting incumbents’ financial capacity to sponsor violence. While some can afford to invest in expensive, long-term relationships with violence perpetrators, others are constrained to rely on more affordable and ad-hoc recruitment.

Subnational incumbents financially invest in ‘renting’ political violence perpetrators, such as militants, militias, paramilitaries, vigilantes and ordinary citizens, to manipulate elections. For instance, politicians in Kenya and Ghana recruit citizens for electoral violence (Bob-Milliar, 2014; Klaus, 2020), while those in Nigeria and Pakistan hire armed groups such as the Ijaw Youth Council and the PAC, respectively (Siddiqui, 2023; Turnbull, 2021b). Drawing on Weinstein (2006)’s argument that rebel leaders use resources to incentivize recruitment into rebel groups, I argue that incumbents use rents to incentivize perpetrators for electoral violence.

High rents enable incumbents to hire expensive but electorally valuable armed groups, while low rents limit them to prioritize affordability, relying instead on ordinary citizens. I define armed groups as organizations that routinely engage in violence for profit during and outside elections. These armed groups leverage electorally valuable qualities relevant for demobilizing incumbents’ rivals to accumulate high financial benefits from incumbents. First, their local integration and structured organization provide extensive connections relevant for navigating local areas and identifying electoral targets (Staniland, 2015b). Second, armed groups have violent reputations that are instrumental in instilling fear and exercising control through the intimidation of diverse targets, including opposition candidates, voters, security agents and election officials (Iwilade, 2014; Rivera et al., 2025). Third, their established structure and reputation afford them a degree of autonomy, enhancing their bargaining power with incumbents. To clarify, the argument does not preclude citizens’ participation in high-rents areas, rather it highlights incumbents’ preference to contract armed groups.

In contrast, low-rents incumbents rely on ordinary citizens hired for ad hoc execution of violence. Citizens are typically drawn from the incumbents’ support base of co-partisans and co-ethnics. While some may have affiliations with armed groups, their recruitment in low-rents areas is mainly individual rather than organized. I argue that their lack of organization impacts their ability to effectively carry out political violence. First, citizens’ ad hoc structural recruitment limits extensive influence, only allowing for localized targeting due to familiarity with their immediate environment. Second, lacking violent reputations, ordinary citizens are perceived as non-threatening and are less effective in coercing other voters. Third, without established structures or reputations, ordinary citizens lack autonomy, leading to dependency on incumbents. This dependency restricts their ability to negotiate higher payments, allowing incumbents to offer only minimal financial incentives.

The argument has implications for the duration of incumbents’ relationships with political violence perpetrators. While high-rents incumbents maintain long-term relationships with armed groups, low-rents ones privilege short-term arrangements with citizens. While high-rents incumbents maintain relationships through territorialized illicit economies, securing the loyalty of armed groups by granting them control over criminal networks (Albarracín et al., 2025; Trejo and Ley, 2020), limited resources in low-rents areas often preclude sustained ties with citizens.

I derive the following expectation:

(i) Incumbents in high-rents areas are more likely to hire armed groups compared with those in low-rents areas.

## Case selection

Nigeria is a relevant case for several reasons. First, Nigeria is dependent on oil rents, and plagued with the resource curse manifesting in the form of corruption, weak institutions and bad governance (Mehlum et al., 2006; Ross, 1999). Second, its fiscal decentralization, based on an uneven rents-sharing formula, directly allocates rents to subnational governments, giving incumbents privileged access. However, while states receive a fixed percentage of rents in the form of federal allocations, some receive additional allocations based on factors such as population and crude oil presence, leading to an uneven distribution (Iledare and Suberu, 2010). Third, Nigerian elections are characterized by violence, such as clashes, intimidation, political assassinations and attacks (Omotola, 2010a; Oyewole and Omotola, 2022), sponsored by regional governors, especially influential ones (LeBas, 2013; Turnbull, 2021b).

I probe the plausibility of the argument using four Nigerian states: Lagos, Rivers, Nasarawa and Plateau. Plausibility probes allow for proving the details of a particular case to shed light on a broader theoretical argument (Levy, 2008). I selected states as units of analysis because unlike local governments, they possess fiscal autonomy over rents. This probe compares subnational patterns in Lagos, Rivers, Plateau and Nasarawa states.

The cases were selected to capture variation in rents (Table 1), but the selection process has limitations, reflecting the common challenge in qualitative research of assuming complete information about cases. During and after data collection, it became clear that not all alternative explanations could be excluded, resulting in an imperfect research design. To navigate this, I use a comparative case design, comparing states that share similarities in ethnic heterogeneity, party identity and availability of violence perpetrators to rule out such alternative explanations. The cases do not present a comprehensive test of the argument; rather, they offer evidence that it is worth exploring further.

**Table 1.** Overview of the cases and alternative explanations.

	Lagos	Rivers	Plateau	Nasarawa
Rents	High	High	Low	Low
Violence perpetrator	Armed groups (*National union of transport workers*)	Armed groups (*Cult groups*)	Ordinary citizens (*Party supporters*)	Ordinary citizens (*Ethnic/party supporters*)
Electoral violence	High	High	Low	Low
***Alternative explanations***				
Availability of violence perpetrators	High	High	High	High
Ethnic heterogeneity	High	Higher	Higher	High
Outbidding	Higher	Higher	High	High
Party identity	All Progressives Congress (APC)	Peoples Democratic Party (PDP)	Peoples Democratic Party (PDP)	All Progressives Congress (APC)

Table 1 compares four Nigerian states across key variables. It shows that Lagos and Rivers, both high-rents states, experience high levels of electoral violence and rely on armed groups as perpetrators. In contrast, Plateau and Nasarawa, with low rents, experience low violence and rely on ordinary citizens. Importantly, alternative explanations such as perpetrator availability, ethnic heterogeneity and outbidding are similarly high across all cases, suggesting that variation in rents, rather than the alternatives, best explains variation in perpetrator type.

## The data

I evaluate variation in violence perpetrators using original data collected during eight months of fieldwork in 2022–2023. My argument expects variation in perpetrator types as a function of rents. The data comes from 128 semi-structured interviews (averaging 45 min)^4^ with local politicians, journalists, civil society members and ordinary citizens in Nasarawa, Plateau, Rivers and Lagos states, supplemented by observer reports and newspaper articles. Respondents were selected using purposive and snowballing sampling techniques.^5^ I analyse the interviews thematically, identifying reoccurring themes through coding done on *Atlas.ti*.

I operationalize rents using revenue derived from crude oil in Nigeria. These revenues, referred to as federal allocations, largely comprise oil exchange earnings (National Bureau of Statistics, 2021). I use qualitative interviews to examine the relationship between political violence perpetrators and incumbents.

### Qualitative data summary

The qualitative data is based on two fieldwork trips conducted in Lagos, Rivers and Nasarawa states over a period of eight months.^6^ I connected with reputable civil society organizations (CSO) – YIAGA Africa, RULAAC, Enough is Enough, Stakeholder Development Network – and their assistance was very helpful in finding respondents.

To avoid leading and sensitive questions, the interview guide was reviewed by university colleagues, Nigerian experts and local fieldwork contacts. I strictly adhered to the interview guide, making sure to maintain similar interviewing standards. Interviews with CSO members and local journalists focused on violence perpetrators’ organizational structures, recruitment patterns, and their relations with incumbents. Discussions with local politicians and citizens examined the impact of rents on incumbents’ financial capacity and associations with violent groups.

Unable to access armed groups, interviews with journalists and CSO members were relevant alternatives, offering valuable perspectives on armed groups’ activities. Journalists and CSO members are knowledgeable about armed groups due to contacts from interviewing them or from eyewitness reports. Interviews followed ethical standards, prioritizing respondents’ anonymity and privacy.^7^

**Figure 1.**
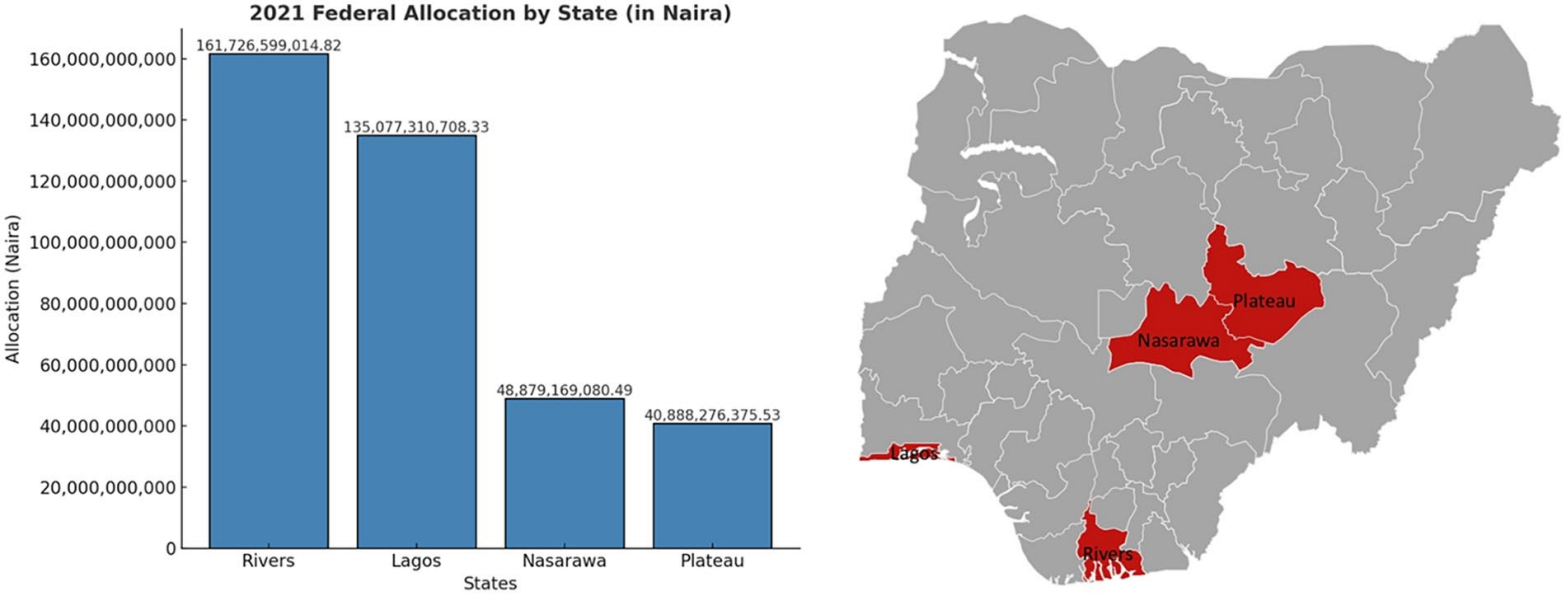
Rents and electoral violence in Nigeria (2019), and a map of Nigeria. Sources: Nigeria Watch, National Bureau of Statistics and Map chart.

As shown in the scatterplot in Figure 1, rent is positively correlated with electoral violence. High-rents states such as Rivers and Lagos are highly violent, while low-rents states such as Nasarawa and Plateau witness lower levels of violence. Beside the scatterplot is a map visually illustrating the geographical locations of the cases.

**Figure 2.**
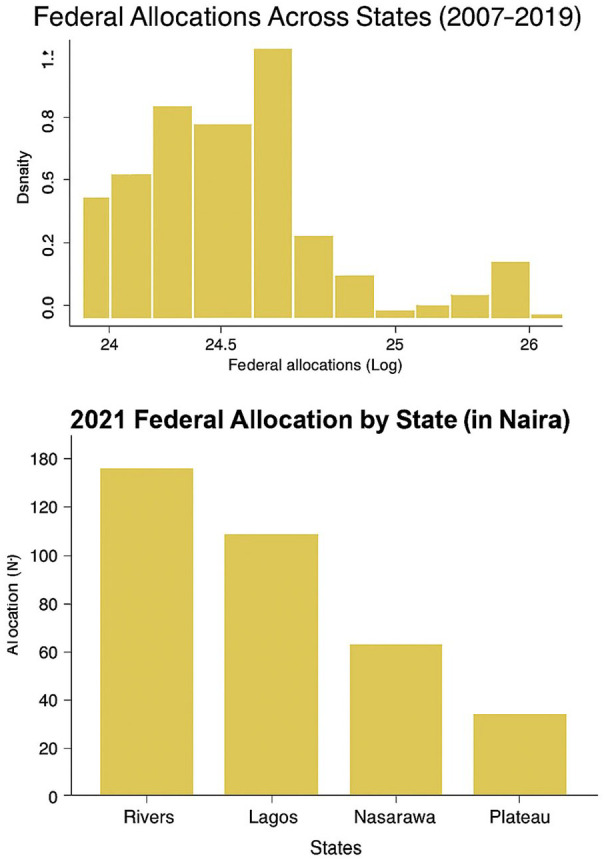
Federal allocation (rents) distribution. Source: National Bureau of Statistics (2021).

Figure 2 presents two graphs illustrating the distribution and variation of federal allocations across Nigerian states. The top histogram shows the distribution of federal allocations from 2007 to 2019 (log-transformed), revealing that most states received moderate allocations clustered around the 24–24.5 range. The bottom bar chart highlights allocations in 2021 for four selected states – Rivers, Lagos, Nasarawa and Plateau – showing a distinct variation: Rivers and Lagos received substantially higher allocations compared with Nasarawa and Plateau.

## Findings from the case studies

### Lagos state

Lagos, Nigeria’s commercial hub, is the most populous state, with over fifteen million residents (Lagos state government, 2023). It has an ethnically heterogenous population, with the Yorubas as the major ethnic group. Given its high population and thriving economy, Lagos receives large federal allocations, leading to the accumulation of high rents. Since 1999, the All Progressives Congress (APC)-led government, though rebranded under different names, has maintained power. However, Lagos elections have been characterized by violence (Abe, 2023).

Due to the availability of high rents, rent-seeking incumbents have sponsored violence against opposition parties including the Peoples Democratic Party (PDP) and the Labour Party (LP). According to a civil society election observer,

You find here structures of capital or is it capital production? What am I trying to say here is the money here is huge, availability of resources is huge. So what am I driving at, the amount, this is my reading that an elective officer will spend in Lagos will be relatively more than his or her other counterparts will spend in other parts of the country, the stakes are high.^8^

The extensive rents in Lagos not only incentivize incumbents but also enhance their spending capacity relative to other states. Hence, determined not to lose access to rents, incumbents have mainly aligned with the militant wings of the National Union of Road Transport Workers (NURTW) for electoral violence despite the availability of other armed groups.^9^ Established in 1978 to protect the interests of road transport workers, the NURTW, hereafter referred to as the Union, has significantly deviated from its original mandate (Fourchard, 2023; Omobowale and Fayiga, 2017). Currently, it operates as a mafia-like organization involved in extortion, violence and other criminal activities (Adejumo, 2016; Agbiboa, 2022).

The APC’s alignment with the Union is material-based, premised on the exchange of financial incentives for support.^10^ As noted by an APC politician, the Union is the *instrument* the APC uses for *violence*.^11^ He noted that the collaboration between the Union and the APC is largely material, premised on the exchange of material benefits for the supply of violence. Although respondents did not know exact Union wages for violence, they provided estimations.

According to this respondent,

It’s money they give them. *(APC party leader)* pays millions, do you know that (NURTW chairman) who is simply an *agbero (tout)*, all his children are studying in the United States, just a motor park tout. He gets the best medical attention, he travels, where does he get the money from? So they benefit a lot in terms of money, they also get properties and all that.^12^

Citing multimillion-naira payments and the lavish lifestyles of Union executives, the respondent highlights the significant financial rewards derived from the Union’s collaboration with the APC. Such lifestyles sharply contrast with the expectations for working-class individuals, revealing the profitability of these alliances. Respondents noted that these benefits are distributed within the Union, fostering loyalty and dependency. In return, Union members, known as *agberos* (local thugs), deploy violence to demobilize opposition parties. Although the Union is technically available for hire, opposition parties including the PDP and LP often lack the financial resources to compete with the APC.^13^

Respondents highlighted that incumbents align with the Union due to its strategic relevance in Lagos.^14^ This strategic relevance aids *agberos* to effectively carry out violence. First, the Union’s extensive structure enables *agberos* to identify rival voters. Present in the numerous bus stations across Lagos, the Union is deeply embedded in the state. This localized presence allows *agberos* stationed in these areas to become familiar with their surroundings, enabling them to recognize the partisan identities of voters within their areas of influence.^15^

Second, *agberos*’ notoriety for violence enhances the violent reputation of the Union (Fourchard, 2023; Lambo, 2022). Leveraging their reputation, *agberos* effectively instil fear during elections through voter intimidation, ballot box destruction, and attacks on rival candidates and election officials. Respondents recounted incidents from the 2023 elections, where *agberos* not only intimidated opposition voters but also targeted voters in incumbent strongholds whom they perceived as unlikely to support the ruling party. Another respondent recounted that after ignoring warnings and attempting to vote, he was flogged by *agberos* at the polling station and prevented from voting.^16^ News of such harassment quickly spread, resulting in low voter turnout in opposition strongholds.^17^ Supporting these accounts, news reports revealed that during the 2023 gubernatorial elections, the Union chairman openly threatened opposition voters, warning them against participating in the election (Inyang, 2023).

Appreciative of the Union’s electoral value, incumbents heavily invest in long-term alliances. Respondents highlighted that the APC’s dominance in Lagos politics is largely sustained through these partnerships, with the Union employing violence to demobilize rivals and suppress anti-party resistance even outside election periods.^18^ Recognizing the Union’s utility, the APC provides financial support, covering operational costs and enabling the Union to exploit Lagos’s high-rents environment. Such exploitation manifests in the form of extortion and other crimes.^19^ Leveraging Lagos’s lucrative transport sector, the Union coercively imposes fees on commercial motorists, often operating beyond its official mandate. Under the protection of incumbents, Union members engage in these activities without fear of legal consequences.^20^

## Rivers state

Rivers has a diverse ethnic population, mainly consisting of the Ikwerres, Ogonis and Ijaws, with over eight million residents living in its Upland and Island regions (Rivers State Government, 2023). It has extensive natural gas and crude oil reserves, accounting for over 40% of Nigeria’s oil and gas production (Rivers State Government, 2023). As an oil-rich region, it receives additional allocations from oil export earnings, making Rivers a high-rents state. However, the presence of oil has fuelled agitations over resource autonomy, leading to violent clashes with the federal government and the rise of militancy (Ebienfa, 2011). This competition over resources extends to subnational politics, where rent seeking shapes political competition. Describing the high rents, according to this election observer,

Rivers State is an oil-producing state and it is also a state that the Internally Generated Revenue (IGR) is very high. And, Rivers state is one of the states that is very prominent and well to do when it comes to economy in Nigeria . . . we have a lot of national investments here in Rivers state from Nigerian National Petroleum Company (NNPC) there are a whole of federal government investments here in Rivers state. So the person who becomes the governor is also controlling the taxes from the companies in Rivers state and it is something that everybody is eyeing.^21^

Given the availability of high rents, observer reports indicate that violent rent seeking fuels contentious politics between ruling and opposition parties (European Union Election Observer Mission Nigeria, 2019). A Human Rights Watch (HRW) report highlights how Rivers’ rents have been diverted to sponsor violence and insecurity for ruling party politicians (Albin-Lackey, 2008). Similarly, the ruling party PDP, like the APC in Lagos, hires armed groups to demobilize opposition parties.

According to a local politician,

Sponsorship of this violence, thuggery, and voter intimidation was heavily funded by the incumbent . . . of course, it is difficult for opposition members to fund violence because it is actually expensive and so they do not do it on their own or they do not do it alone, they of course outsource. There are some merchants of violence in town, so they procure these persons to executing in camps so that is why the violence becomes so pronounced. It is expensive because it entails the doling of ‘free money’ money from government coffers which is likely unaccounted for which is a direct resource of the products of corruption that’s why it is easier for the ruling party to fund such illegalities.^22^

Although the financial costs of sponsoring violence are high, a respondent noted that incumbents conveniently outsource violence to ‘merchants’ using funds from government coffers. According to other respondents, these merchants are known as cultists. Established in the 1950s as non-violent fraternities promoting Pan-Africanism, these cults gradually turned violent (Nyiayaana, 2011; Rotimi, 2005). Hence, recognizing their violent potential, incumbents began hiring them for electoral violence (Eguavoen, 2008; Ogele et al., 2020; Rotimi, 2005). Examples include Icelanders, Greenlanders, Deewell and Deebam.

Multiple respondents estimate that incumbents spend millions of naira, or its equivalent in dollars, to hire armed groups. In addition to these payments, incumbents purchase sophisticated and expensive weapons for cult groups. For instance, prior to the 2003 elections, former Governor Peter Odili funded and armed cult groups to secure a decisive victory for the PDP (Albin-Lackey, 2008). An opposition politician explained that opposition parties’ inability to hire cults is not due to a lack of interest but rather limited financial resources, as incumbents largely control the funding needed to sponsor violence.^23^

Respondents noted that incumbents hire numerous cult groups, leveraging their electorally valuable qualities to effectively carry out violence. First, cults have established structures, with clearly defined spheres of influence, allowing them to control specific areas within local communities.^24^ Second, their violent reputations, gained from crime and brutal killing methods, make them effective for demobilizing opposition voters.^25^

According to one respondent, cults engage in sporadic shootings before elections to instil fear.^26^ Multiple respondents affirm that such strategies are largely effective in reducing opposition voters’ turnout. Recognizing their electoral utility, incumbents maintain long-term relationships with cult groups. Respondents highlighted that incumbents rely on cults even during non-electoral periods to suppress opposition activity and prevent rivals from gaining influence.^27^ Cults use violence to suppress opposition uprisings, thereby helping incumbents to consolidate control.^28^ To secure their loyalty, incumbents provide routine payments, allowing cultists to exploit Rivers state’s high-rents environment to generate income. Hence, cults engage in illicit activities, including sea piracy and oil bunkering, under the protection of incumbents. ^29, 30^

### Nasarawa state

Nasarawa has a diverse population of less than three million, mainly comprising the Wandara, Alago and Eggon (Nasarawa State Government, 2023). With agriculture as the mainstay, Nasarawa’s economy ranks low compared with other states (Ejechi, 2024). Coupled with its low population and lack of crude oil, Nasarawa receives fewer federal allocations compared with other states. Despite slow economic progression, news reports show that Nasarawa has been plagued with farmer-herder crises and banditry (Agwam, 2024; Nwankwo, 2024).

The political landscape of Nasarawa state is characterized by the absence of one-party dominance, with power rotating among parties including the PDP, APC and Congress for Progressive Change (CPC). Despite the low rents in the state, politicians as in other parts of Nigeria engage in violent forms of rent seeking, using violence against opposition parties.^31^ Such violence is mainly concentrated in highly populated local government areas such as Awe.^32^ However, unlike high-rents areas, incumbents hire ordinary citizens drawn from party networks to execute violence.^33^

According to this local politician,

I think Nasarawa is a little bit different from Rivers state, when you see thugs, political thugs they are members of that political party, they recruit their members. If you go to the collation centre you will see PDP thugs, you will see APC thugs, they are members of that political party they perpetrate this evil . . . and at the end the only thing they will give them is 10k ($10), 5k ($5) nothing much unlike that of Rivers if you have done that you are in money they can give you billions and millions of naira but here you can’t get more than 20k ($20).^34^

As the respondent explains, incumbents in Nasarawa rely on party supporters to perpetrate violence. However, these supporters are paid modest sums, such as $20, which is substantially lower than the millions of naira earned by armed groups in high-rents states such as Rivers. Respondents noted that party support groups organized for electoral violence are ad hoc in nature.^35^ First, they lack organizational structure beyond their local communities, relying on local knowledge to identify rival voters within their immediate surroundings.^36^ Second, respondents noted that party supporters lack the fear factor associated with armed groups, as they are often known to others as neighbours and friends.^37^ Third, due to their lack of structure and violent reputation, these supporters have limited autonomy to negotiate for higher payments from incumbents.^38^

When asked why incumbents in Nasarawa prefer citizens over available armed groups, respondents attributed the choice to cost considerations. As one respondent explained, ‘It is difficult to have thugs on standby . . . the reason is that fueling these thugs is cost-expensive . . . it is quite expensive rather.’^39^ Respondents explained that because hiring and maintaining armed groups is financially burdensome, incumbents rely on their support base.^40, 41^

To avoid maintenance costs, incumbents maintain short-term relationships with party support groups, disbanding them as violent units after elections. This allows incumbents to avoid the financial responsibility of sustaining these groups between electoral cycles.^42^ In Nasarawa’s low-rents environment, there are few lucrative opportunities for citizens to exploit for additional revenue.^43^ Hence, after elections, citizens typically return to their regular livelihoods, such as hunting, farming, petty crime or unemployment.^44^

### Plateau state

Plateau, with a population of over three million and major ethnic groups including the Berom, Anaguta and Fulani, is predominantly an agricultural state (Plateau State Government, 2023). This diversity has made the capital city, Jos, a hotspot for communal conflicts driven by conflict over ‘indigene’ rights and political representation (Krause, 2011). Beyond the city, occasional farmer-herder clashes over grazing land further exacerbate the volatility in rural areas (Olufemi, 2021).

Plateau has a fluid partisan identity, characterized by power alternations between the PDP and APC. Economically, it ranks 33rd out of Nigeria’s 36 states, making it a low-rents state (BudgIT, 2022). As this respondent explains,

We do not have money here but remember that if you are the chief executive, you control resources. We have what we call the subvention that comes from the federal government that goes from federal to state and state to local government. That is why some people will want to be leaders so that they can control the subvention. And we cannot totally say there is no money on the Plateau but of course it cannot be compared to Lagos and many other states . . . so even though we don’t have money per se this is why people will be ready to die because they know there is a benefit attached to these positions.^45^

Despite the limited rents, respondents noted that politicians still compete for financial benefits attached to political offices.^46^ Although numerous violence perpetrators are available, respondents noted that incumbents rely on ordinary citizens. This perspective aligns with an HRW report (Human Rights Watch, 2005) that identifies citizens, not armed groups, as the main violence perpetrators hired by political sponsors. As noted in the report and journalistic accounts, politicians incentivize unemployed youths in Plateau lacking strong ideological beliefs with the promise of money (Oduah, 2015). According to a local journalist,

Giving them five naira or ten naira and giving them all kinds of drugs and drinks given to them, and they are ready to kill. So, the foot soldiers are usually these young boys who don’t have anything doing and the politicians who are the perpetrators now see them as already made instruments in their hands so they want to use them by all means.^47^

As the respondent explains, the incumbent in Plateau, unlike counterparts in the Niger Delta, relies on ethnic groups rather than cult groups to perpetrate violence. While the exact costs paid to citizens are unknown, he implies that hiring citizens is inexpensive, citing low sums as examples. Aware of their preference for immediate gratification, incumbents incentivize these individuals with small amounts of money, hard drugs and alcohol to carry out violence.^48^ Unlike in Nasarawa, where perpetrators are largely party supporters, respondents in Plateau noted that while perpetrators are mainly co-ethnics of the incumbent, their targets are often aligned along party lines.^49^ This reflects the state’s salient ethnic divides, where indigenous groups typically support the PDP, while settlers align with the APC.

These ordinary citizens are organized for electoral purposes. First, ethnic support groups are loosely organized for electoral violence. However, they leverage their knowledge of party identities to localize violence, targeting rival voters. As this respondent claims, ‘You should be rest assured that members of the rival party they will come to your house, dance, break your windows and it will get violent. It has happened to me, my windows have been broken.’^50^ Second, citizens have no fear factor and are known by other voters by their ethnic affiliations.

In Plateau, the salient ethnic divides made unpacking the mechanisms of electoral violence particularly challenging. Respondents, deeply embedded in the communal conflicts, often shifted conversations from electoral violence to broader communal tensions, making it difficult to keep interviews focused on electoral violence. Despite such challenges, I was able to identify patterns consistent with the recruitment of citizens for violence. However, I did not find sufficient evidence to support the maintenance cost aspect of the theory.

Table 2 summarizes the case comparisons and main findings from the four states, which I discuss and analyse in the next section.

**Table 2.** Summary of case comparison and findings.

State	Rents	Perpetrator	Recruitment strategy	Maintenance investment	Autonomy	Outsourcing risks	Perpetrator income source
Rivers	High	Cults	Strategic local relevance Cults with extensive influence	Yes (long-term relationships)	High independence	High – Occasional loss of control, rival groups used for retaliation	Large sum of money Oil bunkering, piracy
Lagos	High	Union	Strategic local relevance Union controls transport networks	Yes (long-term relationships)	Moderate	Lower – Partial dependency allows some control	Large sum of money Transport extortion
Nasarawa	Low	Ordinary citizens	Co-partisans	No (short-term/ad hoc)	Low	Minimal – Citizens have low bargaining power	Small stipends, groceries, alcohol
Plateau	Low	Ordinary citizens	Co-ethnics	No clear evidence	Low	Minimal – Citizens have low bargaining power	Small stipends, groceries, alcohol

## Comparing the cases

Interviews and observer reports confirm that rents are relevant for hiring and maintaining violence perpetrators. Findings reveal a significant cost difference: armed groups earn millions of naira, while ordinary citizens receive only a few thousand. High-rents incumbents in Rivers and Lagos utilize their rents to cover both recruitment and maintenance costs, whereas Nasarawa and Plateau cover only recruitment expenses. I compare the cases along two key dimensions: recruitment and maintenance costs.

First, high-rents incumbents recruit armed groups based on their strategic local relevance, despite occasionally using ordinary citizens. In Rivers and Lagos, incumbents hire distinct types of armed groups due to their strategic relevance in each state. Cults and the Union share common features such as established structures, violent reputations and autonomy, yet their utility varies by context. In Lagos, the Union’s value is linked to its control over the broad commercial transport networks, making it a viable option for extensive local influence.^51^ In contrast, cults, confined to remote areas in Lagos, have extensive influence in Rivers. Cults’ local presence in the Upland and Island regions makes them more appealing to incumbents compared with the Union’s limited reach in the state’s Island areas.^52^

Incumbents must navigate the risks associated with outsourcing violence. As Siddiqui (2022) explains, outsourcing can provide short-term benefits but introduces risks, such as losing control over the armed groups. My findings indicate that incumbents’ control over these groups depends on their level of autonomy. In Lagos, the Union demonstrates proxy-like autonomy, functioning as entities separate from the state but connected to ruling party networks (Staniland, 2015a). The Union’s reliance on government approval to exploit the public transport sector for revenue allows incumbents to leverage this partial dependency to maintain a degree of control over them. Given their mutual utility, respondents were optimistic that strained relations between the two are highly unlikely.^53^

Similar to the Union, cults exercise autonomy vis-a-vis incumbent, sharing similar trajectories with independent armed groups with their own command structures, and logistics (Staniland, 2015a). However, their full autonomy increases the risk of losing control, as their income generation is not entirely dependent on incumbents.^54^ Cults generate revenue from illicit activities such as sea piracy and hostage taking, which fall outside the scope of incumbents. While incumbents protect them from prosecution, this independence has occasionally led to tensions. A respondent cited instances where strained relations between incumbents and cults led to incumbents using state security agents or rival groups to eliminate them.^55^

In contrast, incumbents in low-rents states trade off availability for affordability. In Nasarawa and Plateau states, incumbents align with ordinary citizens despite the availability of armed groups.^56^ Ordinary citizens align with dependent groups, which Staniland (2015a) describes as fully reliant on the government for sponsorship, resources, power and influence. Citizens in Plateau and Nasarawa lack autonomy to demand higher payments, settling for lesser amounts of money, groceries, alcohol and narcotics.^57^

Second, high-rents incumbents financially invest in maintaining long-term relations. In contrast, low-rents incumbents avoid maintenance costs by investing in ad hoc relationships. Although I did not find sufficient evidence in Plateau, in Nasarawa incumbents avoid routine payments by establishing short-term relations with citizens.^58^ Although high-rents incumbents can afford routine payments, they often allow armed groups to exploit the state’s high rents by generating income through illicit activities. In Lagos, the Union exploits the lucrative transport sector through extortion, while cults in Rivers engage in oil bunkering and sea piracy. Respondents in both states noted that long-term relationships with armed groups are beneficial for winning elections in high-rents states.^59^ Thus, one-party dominance in both states suggests that connections with armed groups are essential for consolidating party control while its absence in low-rents states suggests citizens’ inability to do the same. Consistent with Wahman and Basedau’s (2015) findings that incumbents in oil-rich areas limit competition from opposition candidates more effectively than those in other regions (Wahman and Basedau, 2015), my findings suggest that high-rents incumbents consolidate power by using rents to hire armed groups, thereby limiting competition.

An additional fieldwork finding reveals the insignificance of audience costs for violence. In Nigeria, widespread impunity allows politicians to remain largely unconcerned about costs, as incumbents and violence perpetrators are rarely prosecuted for electoral violence. This perception is supported by an HRW report, highlighting the weaknesses of Nigerian institutions to punish violence perpetrators for past election-related offences (Human Rights Watch, 2023). When discussing voters’ responses, respondents in Rivers and Lagos noted that people fear armed group violence, while in Nasarawa and Rivers, they push back against citizen violence.

## Alternative explanations

In this section, I address the alternative explanations highlighted in Table 1, such as availability of violence perpetrators, ethnic heterogeneity, outbidding and incentives. The most plausible alternative explanation for the variation in electoral violence perpetrators is the availability of violent actors. This perspective aligns with scholars who argue that in the absence of ideologically compatible groups, incumbents may collaborate with ideologically incompatible ones, leveraging the available supply of violent actors to perpetrate electoral violence (Staniland, 2015b; Sterck, 2020). However, the presence of violence perpetrators in both high- and low-rents areas suggests that availability is a necessary but insufficient condition to explain the variation. While cult groups and transport unions exist in all states, they are not utilized for electoral violence in low-rents states. For instance, the Union operates nationally, and cults are present on public university campuses across the country. Additionally, reports from Reuters (2023) affirm the presence of armed groups, including bandits and herder-militias, in Plateau state. However, such groups primarily engage in non-electoral violence, such as raids and ambushes on local communities. Highlighting this pattern, Nwankwo (2024) illustrates that spikes in farmer-herder crises in North-Central states such as Nasarawa and Benue occur outside electoral years (Nwankwo, 2024).

A second explanation attributes the choice of violence perpetrators to ethnic heterogeneity. Scholars argue that politicians exploit divisive ethnic narratives to polarize citizens and select perpetrators based on ethnic affiliations (Birch et al., 2020; Klaus, 2020; Wilkinson, 2004). However, evidence from Nigeria challenges this claim. This explanation is invalidated by the case selection. The selected states are ethnically heterogenous, yet incumbents in Lagos and Rivers mainly rely on armed groups. For example, former Rivers governor Peter Odili, despite not being Ijaw, hired the Ijaw Youth Council for violence (Turnbull, 2021a, 2021b). Similarly, the Yoruba-led APC government in Lagos relies on the ethnically diverse Union. In high-rents, ethnically homogeneous states such as Oyo, governors rely on the Union rather than ordinary citizens for violence (Omobowale and Fayiga, 2017). These findings indicate that factors such as financial capacity, rather than ethnic polarization alone, significantly influence incumbents’ selection of violence perpetrators.

A third explanation suggests that incentives, rather than financial capacity, drive incumbents to invest in violence perpetrators. While incentives are undeniably relevant, without financial capacity, incumbents face limitations in sponsoring violence. Evidence from Nigeria shows that variation in capacity is more pronounced than in incentives. Nigerian politicians are largely motivated by rent seeking, using violence to undermine rivals (Abah and Nwokwu, 2015; Smah, 2008; Yusuf, 2019). This is evident in states such as Kogi, Kwara and Ekiti, where incumbents sponsor violence even with low rents. For instance, during the 2003 Kwara state elections, incumbent governor Mohammed Lawal and his former godfather, Bukola Saraki, following a political rift, each mobilized supporters for electoral violence (Luqman, 2010). While incentives are important, they are insufficient to explain variation in violence perpetrators.

The fourth explanation emphasizes outbidding, proposing that candidates’ personal wealth, particularly among non-incumbents, can be sufficient to hire violence perpetrators. This suggests that neither incumbents nor challengers necessarily depend on rents to fund electoral violence, implying rents alone cannot explain financial capacity differences. However, this assumes comparable financial strength between incumbents and opposition candidates. Given the high costs of electoral campaigns (Samuels, 2001), expenditures often strain candidates’ finances, with incumbents typically outspending challengers (Sudulich and Wall, 2011).

In Nigeria, opposition candidates often lack the resources to outbid incumbents in hiring violence perpetrators. While candidates may rely on party strongmen or godfathers for sponsorship, these godfathers primarily use politics to control state resources and consolidate power (Omotola, 2007). Although godfathers maintain private armed networks, these are closely tied to ruling party structures, with rents being necessary for sustaining them. Consequently, godfathers in high-rents states generally possess greater financial capacity than those in low-rents states, further highlighting the impact of rents in enhancing financial capacity (Albin-Lackey and Rawlence, 2007).

The final explanation relates to party strategies, suggesting that their organizational capacity shapes their outsourcing choices – either to citizens or armed groups (Siddiqui, 2023). If valid, stronger parties are likely to hire armed groups, while weaker ones would rely on citizens. However, in the Nigerian case, parties are weak, unstable and poorly institutionalized (Omotola, 2010b), using similar strategies across contexts. For instance, APC and PDP incumbents in Lagos and Rivers hire armed groups, while in Nasarawa and Plateau, despite fluid partisan shifts, citizens remain the main perpetrators. This indicates that party identities are insufficient to explain variation in perpetrators.

I acknowledge that the above alternative explanations are not exhaustive; however, to the best of my knowledge, there is limited evidence to rival the rents argument. One limitation of this study is that the case selection does not allow me to rule out national incumbent agency. While I suspect that national incumbents interfere in subnational politics, the peculiarities of such interference remain unclear and largely undisclosed. Hence, it is difficult to determine whether national incumbents’ agency regarding violence perpetrators impact the observed patterns.

## Conclusion

The study examines how rents shape the variation in political violence perpetrators. The findings highlight the capacity of subnational incumbents to organize electoral violence and the impact of rents in enabling or limiting them. By comparing four cases in Nigeria, a clear pattern emerges: incumbents use material incentives to hire armed groups in high-rents states and ordinary citizens in low-rents ones. The findings have broader implications for generalizability in countries with uneven rents distribution and weak institutions, such as South Sudan, Angola and Venezuela, and in patronage democracies like India where patronage networks provide the infrastructure for mobilizing electoral violence (Berenschot, 2020).

I contribute to work on the joint production of violence by incumbents and non-state actors. Prior work has primarily focused on incumbents’ collaboration with ordinary citizens (Berenschot, 2020; Boone, 2011; Klaus, 2020), outsourcing versus directly perpetrating violence (Siddiqui, 2023), and alliances with armed groups (Matanock and Staniland, 2018; Raleigh, 2016; Staniland, 2015c; Turnbull, 2021a, 2021b, 2024), using different contexts. In contrast, my argument explains why and how subnational incumbents collaborate with distinct types of perpetrators within the same context, holding country factors constant. Additionally, the findings have important policy implications, revealing that while fiscal decentralization aims to promote development, it can also enable subnational incumbents to divert public resources to sponsor electoral violence, highlighting the need for fiscal transparency in governance.

Future research on electoral violence should examine how national and subnational incumbents’ relationships shape patterns of violence. For example, low-rents incumbents aligned with the national ruling party may receive financial support from the centre, enhancing their capacity for violence and potentially surpassing high-rents incumbents. Additionally, further investigation is needed into perpetrators’ targeting patterns. Whether collective or individual, such patterns are relevant for understanding variation in the intensity of subnational electoral violence.

## Notes

1. I define rents as unearned income derived from material resources such as natural resources, foreign aid, and loans (Farzanegan et al., 2018; Sollenberg, 2012). I measure it with fiscal transfers (federal allocations) largely derived from oil rent.

2. I define armed groups as organizations that routinely engage in violence for profit during and outside elections.

3. I describe ordinary citizens as co-ethnic and partisans drawn from the incumbents’ support network.

4. Interview numbers varied significantly based on context. In high-rents states such as Lagos, scheduling differences with respondents posed challenges, leading to an extended stay compared with other locations.

5. With snowballing sampling, respondents recommended by prior respondents were selected based on their experience and knowledge of the research subject.

6. During fieldwork, I visited Lagos and Rivers states on both trips. In Rivers state, I spent a total of two months (March 2022, April 2023). In Lagos state, my initial trip lasted six weeks (April–mid-May 2022) and my second trip spanned two months (3–16 May, 6 June–12 July 2023). My stay in Nasarawa state was shorter (17 May–5 June) as I completed the desired number of interviews within that timeframe.

7. Detailed information regarding ethics can be found in the Online Appendix.

8. Interview, Lagos, local politician. 21 April 2022.

9. Interview, Lagos, local journalist. 20 June 2023.

10. Interviews, Lagos, local journalist, CSO member. 24 April 2022.

11. Interview, Lagos, local politician. 5 April 2022.

12. Interview, Lagos, local journalist. 3 May 2022.

13. Interview, Lagos, election observer. 14 June 2022.

14. Interview, Lagos, local journalists. 19 May 2022.

15. Interview, Lagos, transport worker. 10 June 2023.

16. Interview, Lagos, party supporter. 27 June 2023.

17. Interview, Lagos, party supporter. 1 July 2023.

18. Interview, Lagos, local journalist. 2 July 2023.

19. Interview, Lagos, local journalist. 5 April 2022.

20. Interview, Lagos, local journalist. 10 June 2022.

21. Interview, Rivers, election observer. 4 March 2022.

22. Interview, Rivers, local politician. 7 April 2023.

23. Interview, Rivers, local politician. 14 April 2023.

24. Interviews, Rivers, local politicians, election observer, local journalists. March 2022/April 2023.

25. Interviews, Rivers, local politicians, election observer, local journalists. March 2022/April 2023.

26. Interview, Rivers, election observer. 11 March 2022.

27. Interview, Rivers, local journalist. 4 April 2023.

28. Interview, Rivers, election observer. 2 April 2023.

29. Interviews, Rivers, local politicians, journalists, election observer. 2–26 April 2023.

30. Interviews, Rivers, local politicians, journalists, election observer. 2–26 April 2023.

31. Interview, Nasarawa, election observer. 3 June 2023.

32. Interviews, Nasarawa, local politicians. 22 May 2023.

33. Interviews, Nasarawa, party supporters, election observer. 30 June 2023.

34. Interview, Nasarawa, local politician. 23 June 2023.

35. Interviews, Nasarawa, local politicians, local journalists, election observers. June 2023.

36. Interviews, Nasarawa, local politicians, journalists, CSO members, party supporters. 18 May–5 June 2023.

37. Interviews, Nasarawa, local politicians. 22 May 2023.

38. Interviews, Nasarawa, party supporters. 4–5 June 2023.

39. Interviews, Nasarawa, election observer, party supporter. 30 May–3 June 2023.

40. Interview, Nasawara, election observer. 30 May 2023.

41. Interviews, Nasarawa, election observers, local politicians. 18 May–5 June 2023.

42. Interviews, Nasarawa, election observers, local politicians. 18 May–5 June 2023.

43. Interviews, Nasarawa, election observers, local politicians. 18 May–5 June 2023.

44. Interview, Nasarawa, local politician. 22 May 2023.

45. Interview, Plateau, election observer. 30 May 2022.

46. Interview, Plateau, election observer. 1 June 2022.

47. Interview, Plateau, election observer. 21 May 2022.

48. Interviews, Plateau, local politician. 25 May 2022.

49. Interview, Plateau, election observer. 1 June 2022.

50. Interview, Plateau, party supporter, 27 May, 2022.

51. Interviews, Lagos, local politician. 4 May 2022.

52. Interviews, Rivers, election observer. 11 March 2022.

53. Interview, Lagos, local politician. 17 June 2022.

54. Interviews, Rivers, election observer, local journalists. March, 2022/April 2023.

55. Interviews, Rivers, local journalist. 12 April 2023.

56. Interviews, Nasarawa, election observer, local journalists. 18 May–3 June 2023.

57. Interviews, Nasarawa, Plateau, election observers, local politicians. 18 May–3 June 2023.

58. Interviews, Nasarawa, Plateau, election observers, local politicians. 18 May–3 June 2023.

59. Interviews, Rivers, Lagos, election observer, local journalists. April–May 2022, April–July 2023.
